# Transition From Open to Full Robotic Living Donor Left Liver Graft Procurement for Pediatric Recipients- Experience From a Western Center

**DOI:** 10.3389/ti.2025.15571

**Published:** 2025-12-15

**Authors:** Lucas Pflieger, Xavier Muller, Guillaume Rossignol, Mathias Ruiz, Remi Dubois, Teresa Antonini, Kayvan Mohkam, Jean-Yves Mabrut

**Affiliations:** 1 Department of General Surgery and Liver Transplantation, Croix-Rousse University Hospital, Hospices Civils de Lyon, University of Lyon 1, Lyon, France; 2 Hepatology Institute of Lyon, UMR 1350 PaThLiv, IHU EVEREST, Lyon, France; 3 Department of Pediatric Surgery and Liver Transplantation, Femme Mere Enfant University Hospital, Hospices Civils de Lyon, Lyon, France; 4 Department of Pediatric Hepatology, Gastroenterology and Nutrition, Reference Center for Biliary Atresia and Genetic Cholestasis, Femme Mere Enfant University Hospital, Lyon, France; 5 Department of Hepatology, Croix-Rousse University Hospital, Hospices Civils de Lyon, University of Lyon 1, Lyon, France

**Keywords:** living donor liver transplantation, robotic surgery, donor safety, partial liver graft, pediatric liver transplantation

Dear Editors,Recent recommendations state that robotic living donor partial hepatectomy (R-LDH) does not negatively affect recipient outcomes and provides a higher precision for bile duct dissection [1]. Furthermore, data from a high-volume center for R-LDH show a reduction in donor morbi-mortality compared to the open and laparoscopic approaches [2, 3]. In addition, a worldwide survey from 2023 has shown that the majority of minimally invasive LDH are now performed by a robotic approach (64%) and 41% of the centers performing R-LDH transitioned directly from open to the robotic approach [4]. In light of these recent developments, there is an urgent need to assess the safety of the transition from open to R-LDH in the setting of a low-volume LDH center.

We report all consecutive LDH performed from September 2019 to March 2025 in a high volume tertiary hepatobiliary and liver transplant center from France (>100 liver resections and >100 liver transplantations per year). Of note, all LDH were left grafts allocated to pediatric recipients. The transition from open to R-LDH took place in October 2023 after a 15-year experience with laparoscopic oncological liver surgery and 30 major HPB robotic interventions. Our technique for R-LDH has been previously described [5]. In the open LDH group we performed a supraumbilical midline incision while in the R-LDH group, the graft was extracted by a suprapubic incision. Of note, the technique for parenchymal transection was the same for open and R-LDH and consisted in an irrigated bipolar coagulation without the use of energy devices or CUSA®. The same donor selection criteria were applied to open and robotic LDH. This IDEAL Stage 2a study aims to assess safety of R-LDH in comparison to open-LDH. The primary safety endpoint is the absence of major donor morbidity (CD > II) after 90 postoperative days, based on the Clavien-Dindo Classification (CD). Secondary safety endpoints include graft warm ischemia time defined as the duration from division of the left portal vein to cold flush of the partial graft on the back table, conversion rates, biliary complications and 90-day graft survival. The local ethical review board granted ethical approval of the study.

During the study period, 23 LDHs were performed, with 16 (70%) open LDHs and 7 (30%) robotic LDHs (Table 1). The majority of LDHs were G23 (n = 21, 91%) with one G1234MHV in the open-LDH and one G234 in the R-LDH group. The operative duration of robotic-LDH was significantly longer (356 min vs. 243 min, *p = 0.026*) but intraoperative blood loss was further reduced (50 mL vs. 125 mL *p < 0.001*) and no conversion was required. There was no need for pedicular clamping during parenchymal transection and hilar plate division in both groups. Donor warm ischemia time was <5 min in both groups. At 90 days, there were no CD > II complications in the R-LDH and open group. The R-LDH group had a significantly lower peak INR (1.15 vs. 1.34 *p = 0.025*) without significant differences in postoperative hepatocyte injury (AST: 264 UI/L vs. 428.5 UI/L, *p = 0.222*; ALT: 348 UI/L vs. 765 UI/L, *p = 0.109*) and peak serum bilirubin (17 vs. 23.5 μmol/L, *p = 0.0523*). There was no significant difference in length of hospital stay between the two groups (7 days vs. 7 days, *p = 0.45*). After a median overall follow-up of 25 months, one donor in the open LDH group presented with a symptomatic incisional hernia with the need for a surgical repair (CD IIIb) and graft and recipient survival was 100% in both groups.

**TABLE 1:** Overall donor characteristics and outcomes between open and robotic approach.

	All LDH (n = 23)	Open LDH (n =16)	Robotic LDH (n = 7)	*p*
Preoperative donor characteristics				
Age, y (IQR)	33 (30.5; 38.5)	36.5 (32; 39)	32 (30.5; 32.5)	ns
BMI, kg/m^2^ (IQR)	23 (20.5; 26)	24.5 (20.75; 26.25)	22 (20.5; 22.5)	ns
Operative characteristics				
Graft type: G23, n (%) G234, n (%) G1234MHV, n (%)	21 (91) 1 (4) 1 (4)	15 (94) 0 (0) 1 (6)	6 (85) 1 (15) 0 (0)	ns
Operative time, min (IQR)	272 (235; 322)	243 (223; 300)	356 (278; 372)	*p = 0.026*
Graft weight, g (IQR)	300 (245; 330)	300 (262; 342)	270 (245; 300)	ns
Intraoperative transfusion, mL	0	0	0	ns
Conversion, n	0	n.a.	0	ns
Blood loss, mL (IQR)	100 (50; 175)	125 (95; 212)	50 (0; 50)	*p < 0.001*
Post-operative donor outcomes				
Transfusion during hospital stay, mL	0	0	0	ns
ICU stay, d (IQR)	2 (1; 2)	1 (1; 2)	2 (2; 2.5)	ns
Length of hospital stay, d (IQR)	7 (6; 7.5)	7 (6.7; 7.2)	7 (5.5; 8)	ns
Peak AST, U/L (IQR)	370 (232; 566)	428.5 (284.5; 630.5)	264 (198; 376)	ns
Peak ALT, U/L (IQR)	546 (250; 765)	765 (507.5; 821)	348 (249; 447)	ns
Peak serum bilirubin, µmol/L (IQR)	19 (16; 25.5)	23.5 (17.5; 29.5)	17 (14; 18.5)	*p = 0.0523*
Peak INR, (IQR)	1.3 (1.24; 1.44)	1.34 (1.26; 1.45)	1.15 (1.14; 1.27)	*p = 0.0210*
Peak PLT, x10^9/L (IQR)	173 (155; 200)	167.5 (152.75; 196.5)	185 (175.5; 204)	ns
90 days post-operative complications				
<90 days Clavien Dindo > II, n (%)	0 (0)	0 (0)	0 (0)	ns
<90 days Clavien Dindo I – II, n (%)	5 (22)	4 (25)	1 (14)	ns
Infection (urinary, pulmonary, colitis etc.), n (CD)	4 (CD II)	4 (CD II)	0	ns
Urinary retention, n (CD)	1 (CD II)	0 (0)	1 (CD II)	ns
Opioid use for pain management n (%)	3 (13)	2 (12)	1 (14)	ns
Mortality, n (%)	0 (0)	0 (0)	0 (0)	ns

Abbreviations: BMI, body mass index; IQR, Interquartile, range; ICU, intensive care unit; LDH, living donor hepatectomy.

In this IDEAL 2a study in a small volume LDH center, direct transition from open to R-LDH maintains the open-LDH donor safety standards with the absence of major 90-day donor morbidity. While with R-LDH operative duration was increased by 113 min, there was a significant reduction of intraoperative blood loss, no need for pedicular clamping and no conversion to an open procedure.

In contrast to LT centers from Asia and the Middle East, the experience with LDH in Europe is limited due to availability of deceased donor grafts. Indeed, there are only 15–20 LDH performed every year in France. Furthermore, the team from Brussels, who have one of the largest experiences with LDH in Europe, achieved benchmark outcomes with open-LDH [6]. In their series of 438 open living donor left hepatectomies, the rate of CD Grade III complications was 6% without any Grade IV or postoperative death. In this context, setting-up a LDH program using a minimal-invasive surgical approach is challenging. Thus to launch our LDH program in 2019 we advocated the open approach, in close collaboration with the team from Brussels, with donor safety being the highest priority. After standardizing the open LDH procedure at our center and in the absence of major donor morbidity in the first 16 cases, we decided to directly transition to R-LDH in October 2023. In contrast to laparoscopic LDH, R-LDH allows for a straightforward transposition of operative techniques and steps from the open approach. For example, we applied the same parenchymal transection technique in R-LDH as in open LDH translating into minimal intraoperative blood loss and no need for pedicular clamping. Importantly, despite our small experience, we are convinced that R-LDH offers a real benefit in the dissection of the hilar plate as well as left bile duct division [1]. Although the current series is too small to identify a significant impact on recipient biliary complications, larger series point to a reduction of biliary complications in both donors and recipients with the robotic approach [2].

In conclusion, the transition from open to R-LDH in the setting of a low-volume LDH center can be achieved without compromising donor safety. An extensive experience in minimal-invasive HPB surgery contributes to a safe transition from open to R-LDH.

## Data Availability

The raw data supporting the conclusions of this article will be made available by the authors, without undue reservation.

## References

[1] Hobeika C Pfister M Geller D Tsung A Chan ACY Troisi RI Recommendations on Robotic Hepato-Pancreato-Biliary Surgery. The Paris Jury-Based Consensus Conference. Ann Surg (2025) 281(1):136–153. 10.1097/SLA.0000000000006365 38787528

[2] Broering DC Elsheikh Y Alnemary Y Borja-Cacho D Sturdevant ML Alabbad S Fully Robotic Left Lobe Donor Hepatectomy Is Safer Compared to Open. Ann Surg (2025) 282(1):108–115. 10.1097/SLA.0000000000006705 40135361

[3] Sucandy I Rayman S Lai EC Tang CN Chong Y Efanov M Robotic Versus Laparoscopic Left and Extended Left Hepatectomy: An International Multicenter Study Propensity Score-Matched Analysis. Ann Surg Oncol (2022) 29(13):8398–8406. 10.1245/s10434-022-12216-6 35997903 PMC9649869

[4] Troisi RI Giglio MC Kim J Broering D Cherqui D Wakabayashi G Current Status of Minimally Invasive Surgery for Donor Hepatectomy: A Worldwide Survey (A Joint Initiative of the International Laparoscopic Liver Society and the International Living Donor Liver Transplantation Group). Transplantation (2025) 109:1754–1764. 10.1097/TP.0000000000005423 40484991

[5] Mabrut JY Muller X Mohkam K . Robotic Living Donor Left Lateral Sectionectomy for Liver Transplantation (With Video). J Visc Surg (2025) 162(1):58–60. 10.1016/j.jviscsurg.2024.09.003 39304436

[6] Bonaccorsi-Riani E Daudré-Vignier V Ciccarelli O Coubeau L Iesari S Castanares-Zapatero D Improving Safety in Living Liver Donation: Lessons from Intraoperative Adverse Events in 438 Donors Undergoing a Left Liver Resection. Transpl Direct (2023) 9(9):e1531. 10.1097/TXD.0000000000001531 37636484 PMC10455133

